# Supplementary material for: Learning curve and surgical outcome of robotic assisted colorectal surgery with ERAS program
Source: Sci Rep. 2022 Nov 29;12:20566. doi: 10.1038/s41598-022-24665-w (PMC9709162; doi:10.1038/s41598-022-24665-w)
Supplement: Supplementary file 1 — Supplementary Information 1. [file 41598_2022_24665_MOESM1_ESM.zip › LSC Raw data-Ñ[▒K/092/H_ñw╝╨░O▒Kñσ.pdf]

正式摘要(電子簽章：000000201902961)		
W52 / W52 　☐英文標題(注意:無英文姓名-請補病人基本檔!)	☐【讀取】憑證	

臺中榮民總醫院
出院病歷摘要

轉入號 :　　　　轉出號 :

[redacted]

**(8)**流水編號	
**(9)**入院日期	109/09/23 (2020) 直腸外科 W52 - 153
**(10)**轉科日期	
**(11)**出院日期	109/09/28 (2020) 　住院天數計 5 日

診斷

- 入院 :Rectum cancer, 18cm FAV
- 出院 :R-S juncation cancer , 18cm FAV
  , s/p Laparoscopic Anterior resection on 2020/09/24

主訴

- bloodt stool noted on 2020/9/11

病史

- The 46-year-old male had the history of
  1.RA under medicine control
  2.Sjogren syndrome under medicine control
  He had intermittent blooedy stool since 2020/05. He had went to LMD and hemorrhoid was told. However, much blood was noted on 9/11 and he went to LMD again. Colonoscopy was done and rectum tumor was told. Then, he was referred to our hospital for further treatment. This time, he was admitted for Lap AR.

體檢發現

- Anal inspection:
  Recto digital examination:
  Sigmoidoscopy:
  Anoscopy:
  Abdomen:
  - General appearance :
    - easy looking , conscious clear , without cardiopulmonary distress
    - Vital signs自動帶入date 體溫(BT) 脈搏(P) 呼吸(R) 血壓(BP) 身高(BH) 體重(BW) BMI
      20200923 36.2℃ 98次/分 19次/分 114/74mmHg 159.4cm 78kg 30.7
    - Vital signs: B.P. 114/74 mmHg , P.R. 98 /min , R.R. 19 /min , B.T. 36.2 ℃
  - HEENT :
    - Eyes: no anemic conjunctiva, no icteric sclera, normal EOM
    - ENT: no otorrhea, no rhinorrhea, no erythematous mucosa, no injected tonsil, no deformity
  - Neck: supple, freely movable, trachea midline,no deformity, no engorged jugular vein , no palpable thyroid or lymph node enlargement
  - Chest:
    - Inspection: Grossly normal, symmetric and fully expansion, no accessory muscle used, no operation scar, no spider angiomata, no gynecomastia
    - Palpation: no palpable mass or nodule, no subcutaneous emphysema, no tenderness
    - Ausculation: clear breathing sound
  - Heart:
    - Palpation: no thrill, no heave
    - Percussion: normal heart size
    - Ausculation: regular heart beat, normal S1, S2, no gallop, no murmur

- Extremities: No deformity, full ranged of motion without limitation, no clubbing finger or nail cyanosis, no pitting edema, no ulcer or pigmentation, no obvious skin lesion, no varicose vein, no bone, joint or muscle tenderness

CRS CONDITION

- Karnofsky performance status: (100) Normal, no complaints, no evidence of disease
- PE of Abdomen:
- CEA: 6.31 ng/ml
- Imaging Studies:
- Colonoscope report: tumor 18cm FAV
- Figures:
  1. colon & rectum

手術日期及方法

- 手術日期及方法20200924 Laparoscopic Anterior resection and anastomosis - malignant

住院治療經過

- Operation was done on 2020/09/24. He was under ERAS protocol after operation. Under stable condition, we arranged discharge for him.

合併症

一般檢查

- 累積報告：

DATE	TIME	ALB	TP	BIL.T	BIL.D	ALKP	AST	ALT	LDH	CHOL	TG	HDL-C	LDL	UA	NH3	AMY
1090923	1612		7.1							152	288			6.1		
1090924	1739	3.8		0.44	0.13		18	14								

DATE	TIME	WBC	RBC	HGB	HCT	MCV	PLT	BLST	NEUT	BAND	SEG	LYM	MON	EOS	BAS
1090924	1739	14810		14.0	42.9		248		84.0			10.7	3.9	1.1	0.3

DATE	TIME	NA	K	CL	CA	BUN	CREAT	Mg	P	CRP	ESR	PCT	LIPASE	LACTATE
1090924	1739	142	4.2			14	1.04							

特殊檢查

病理報告

其它

出院時情況

- 3.改門診治療

出院指示

藥名	劑量	劑量單位	用法	頻次	數量	數量單位	現況	天數	單位
Mag. Oxide tab	2	TAB	PO	TID	54	TAB	TH	9	天
Ultracet tab	1	TAB	PO	Q6H	36	TAB	TH	9	天
Plaquenil tab	1	TAB	PO	BID	18	TAB	TH	9	天
Panho tab 40mg	1	TAB	PO	QDAC	9	TAB	TH	9	天

出院計劃

主治醫師 2062F 蔣鋒帆 / 住院醫師 2330E 楊捷儒

正式摘要(電子簽章：**000000201902961)**

W52 / W52

doctorRank:B
